# Physiological change under OsHV-1 contamination in Pacific oyster Crassostrea gigas through massive mortality events on fields

**DOI:** 10.1186/1471-2164-14-590

**Published:** 2013-08-29

**Authors:** Aude Jouaux, Maxime Lafont, Jean-Louis Blin, Maryline Houssin, Michel Mathieu, Christophe Lelong

**Affiliations:** 1 CNRS INEE, BioMEA, Caen Cedex 14 032, France; 2 Biologie des Organismes Marins et des Ecosystèmes Associés (BioMEA), IBFA, SFR ICORE, Université de Caen Basse-Normandie, Caen Cedex 14032, France; 3 Centre de Référence sur l’Huître, Université de Caen Basse Normandie, Caen Cedex 14 032, France; 4 Synergie Mer Et Littoral, Zone conchylicole, Blainville sur mer 50 560, France; 5 Laboratoire Frank Duncombe, Saint Contest Cedex 4 14 053, France

**Keywords:** Crassostrea gigas, OsHV-1, Host response, Mortality, Transcriptome

## Abstract

**Background:**

Massive mortalities have been observed in France since 2008 on spat and juvenile Pacific oysters, *Crassostrea gigas*. A herpes virus called OsHV-1, easily detectable by PCR, has been implicated in the mortalities as demonstrated by the results of numerous field studies linking mortality with OsHV-1 prevalence. Moreover, experimental infections using viral particles have documented the pathogenicity of OsHV-1 but the physiological responses of host to pathogen are not well known.

**Results:**

The aim of this study was to understand mechanisms brought into play against the virus during infection in the field. A microarray assay has been developed for a major part of the oyster genome and used for studying the host transcriptome across mortality on field. Spat with and without detectable OsHV-1 infection presenting or not mortality respectively were compared by microarray during mortality episodes. In this study, a number of genes are regulated in the response to pathogen infection on field and seems to argue to an implication of the virus in the observed mortality. The result allowed establishment of a hypothetic scheme of the host cell’s infection by, and response to, the pathogen.

**Conclusions:**

This response shows a “sensu stricto” innate immunity through genic regulation of the virus OsHV-1 life cycle, but also others biological processes resulting to complex interactions between host and pathogens in general.

## Background

Mortalities of marine bivalves linked to infection by herpes-like virus was first described in 1972 by Farley *et al*. [1] for the eastern oyster, *Crassostrea virginica*. In France, the first mortality of Pacific oysters, *C*. *gigas*, with a herpes virus named OsHV-1 was observed in summer 1991 affecting larvae in hatcheries [2]. Subsequently, this OsHV-1 mortality has been described in both larvae and spat (first year of rearing) [3-5]. This virus has been well described by transmission electronic microscopy and *in situ* hybridization, and detected by both end-point PCR and real-time PCR [6-9]. Its genome has been sequenced from isolated virus particles [10,11].

Massive mortalities have been reported since 2008 on *C*. *gigas* spat and juveniles (second year of rearing) along the French coast. The mortalities extend from spring to summer and seem to be correlated with increased seawater temperature [5,12]. Initially, the Herpes viral agent associated with these mortalities was interpreted as a variant presenting common ancestor with the original form and was named OsHV-1μvar [13]. Thereafter, the presence of OsHV-1μvar and other variants was identified as already present in oyster samples from Normandy collected in 2004 and other variants have been identified in pre-2008 field samples [14]. Recent phylogenetic analysis shows the virus diversity of the different variants of OsHV-1 closed to OsHV-1μvar before 2008 and demonstrates that they are not derived from the reference form [15]. It showed that virus diversity could be observed and that some variants close toμVar could be detected before 2008.

Detection of this virus was based on PCR analysis and numerous samples have documented a systematic association between mortalities and OsHV-1 [16]. More importantly, the role of OsHV-1 in these field mortalities has been proved by experimental infection of Pacific oysters with OsHV-1, either by immersion in presence of experimentally infected oysters or by direct injection of virus particles in the adductor muscle, and by resulting heavy mortalities [17,18].

Netherless, few studies have been performed concerning the physiological response of oyster to herpes virus. Recently, the relationship between host and pathogen has been investigated by Suppression Substractive Hybridisation (SSH) experiments [19] and needs to be well understanding according to physiological response to host against pathogen.

The OsHV-1 presents several particularities like the capacity to infect bivalve different hosts [20-26], but little is known about virus entry mechanisms or virus life cycle. OsHV-1 belongs to Herpesvirus class III, the *Malacoherpesviridae*[10]. This family is composed of two mollusk viruses: OsHV-1 and Abalone herpes virus [27]. In vertebrates, herpes virus strategies into infected host cells have been well studied. Herpes viruses present the particularity of having two life-cycle phases: latency or low production of some viral particles cycle and lytic cycle [28]. The lytic cycle can be induced by stress and involves massive viral replication.

The aim of this study is to understand physiological mechanism involved in pathogens infection and oyster response during massive mortalities observed in the field. Animals were deployed in the different sites before mortalities events. During the beginning of mortality, spat were sampled in infected area and at the same time in sanctuary areas (one natural site and second offshore site), both where no mortality occurred). Percentage of OsHV-1 positive oysters was measured individually by quantitative PCR [8,29]. Physiological aspects of the response to host against pathogens were investigated using microarrays. This methodology has been used for gene expression analysis of specific tissues and of gametogenesis in *C*. *gigas* ([30,31]. In the present study, the response of host to pathogens was investigated in spat according to the mortality event and the level of virus. Some genes were differentially expressed and identification of their potential role is closely related to the annotation of expressed sequence tags (EST) [32].

## Material and methods

### Biological material

Diploid spat of Pacific oysters, *Crassostrea gigas*, were purchased from a hatchery (France Naissain). Spat were reared in a nursery until reaching 6mm shell height and the prevalence of OsHV-1 was measured on 120 animals.

Spat were deployed on oysters beds in April 2011 at three distinct sites, an oyster production area where mortalities on spat was observed in Spring (BL, Blainville sur mer, West Coast of Normandy) whereas to others sanctuary sites (CRIC, Cricqueville en Bessin, East coast of Normandy without oyster production and in an offshore 100m^3^ capacity storage structure deprived from contamination, CAB). Sea water was pumped into the storage structure through a sand filter and was maintained in outdoor reservoir of 25 000 m^3^ before reaching the tank. From the beginning of the study (April) to fall, level of mortality was measured every two weeks in the different sites (depending to tidal accessibility to the field sites). At each sampling, more than fifteen oysters were dissected individually and were sectioned longitudinally, both longitudinal sections included same organs, especially those related to pathogen presence. One section was used to virus quantification and the second to microarray analysis. Samples were frozen in liquid nitrogen and stored at −80°C.

### Virus quantification

Genomic DNA was extracted from one section of each individual using the EpMotion5075 robot (Eppendorf^®^) with the Nucleospin 8 Blood kit (Macherey Nagel^®^) according to the manufacturer’s protocol. The quality and quantity of genomic DNA was estimated on Nanodrop 2000 spectrophotometer (Thermoscientific^®^).

Quantification of viruses was based on the quantitative PCR protocol described by Martenot *et al*. [29] and virus quantity was reported as genomic units (GU) of virus per nanogram of genomic DNA extracted [17,33]. The normalized values were grouped into classes according to log_10_ values: Class 0 = no detectable virus; Class 1 = >0 to 10 GU ng DNA^-1^; etc).

### RNA extraction

Oysters were selected for transcriptomic analysis by microarray based on their viral infection loads during the May mortality episode. Four groups, each comprised of 4 spat having the same viral DNA level, were established. Two were “no virus detected” animals: one from the sanctuary area uninfected wild (CRIC), and one from the isolated tank uninfected offshore (CAB), and two “virus detected” animals were from the oyster grow out site experiencing the mass mortality. The latter two groups consisted of low-virus load individuals (BL) (Log Class 1 with an average 4 GU ng^-1^ DNA) and high-virus load individuals (i) (Log Class 2 with an average 14 GU ng^-1^ DNA).

Total RNA was extracted manually from the second section of each spat in 1 mL TriReagent (Sigma Aldrich^®^) and 100 μL Bromo-Chloro-Propane (Sigma Aldrich^®^). After centrifugation for 15 min at 12000 × g at 4°C, the aqueous phase containing RNA (around 300 μL) was extracted on a silica column with the RNA Nucleospin II kit (Macherey Nagel^®^). The quantity of total RNA was measured on a Nanodrop2000 spectrophotometer (Thermoscientific^®^) and quality was verified using Agilent RNA 6000 Nanokit reagents on the Bioanalyzer platform (Agilent Technologies, Waldbronn, Germany).

### RNA microarray

For each animal, 200ng of total RNA were labelled with Cy3 using the Low Input Quick Amp labelling kit from Agilent technologies according to the manufacturer’s protocol.

Antisense RNA (aRNA) was purified on a column with the RNeasy^®^ plus micro kit (Qiagen). Dye incorporation and aRNA concentration were measured on the Nanodrop2000 spectrophotometer (Thermoscientific^®^) and were congruent to the Agilent’s specifications after synthesis, a labeled aRNA concentration was superior to 2μg and an incorporation of Cy3 higher than 6 pmol Cy3 μg^-1^ aRNA.

One thousand six hundred fifty nanograms of newly synthetized aRNA labelled with Cy3 were hybridized on the custom and validated 4x44K slide containing 31 918 EST [28,30] with the Gene Expression hybridization kit (5188–5242; Agilent Technologies) according to Agilent Technologies’ recommendations. After 16 hours at 60°C (Agilent oven with 10 rpm), slides were washed with the gene expression wash buffer solution (5188–5327; Agilent Technologies). The slides were then scanned on Agilent Technologies G2565AA Microarray Scanner system at 5 μm resolution. Analysis of each spot was made using Feature Extraction software 6.1 (Agilent Technologies), using the default/recommended parameters for the scanning, griding, extraction, correction and normalisation.

### Microarray data analysis

A Principal Component Analysis (PCA) was performed suing the GeneANOVA software [34] to assess the internal consistency of different transcriptional data sets and to obtain the proportion of variance for each principal component.

The intensity of each spot was log_2_-transformed and normalized according to the cyclic Loess technique [35] using the Limma package [36] and R software. Statistical analysis was made using TMeV6.1 (Mev_4_7_4) software [37,38]. ANOVA were performed with high stringency p value of < 0.01 with a Bonferroni’s correction. Hierarchical clustering and K-Means clustering were performed after ANOVA using TMeV on the statistically significant transcripts with Pearson’s correlation as distance matrix and 50 maximum iterations.

### Quantitative PCR

For cDNA preparation, 250 ng of total RNA from each sample (four animals per condition) were reverse transcribed using 200 U of MMuLV-RT (Moloney Murine Leukemia Virus Reverse transcriptase, Promega^®^) in presence of 20 U of RNase inhibitor (RNasin, Promega^®^), 0.5 mM of RNAse free dNTP and in the appropriate buffer (Promega^®^).

Forward and reverse primers were designed using Primer3 software (http://bioinfo.ut.ee/primer3-0.4.0/), avoiding analysis of folding regions. Efficiency of PCR was around 100% determination were defined from a range of cDNA of samples used in this study. The presence of residual genomic DNA was controlled by performing PCR from RNA samples without RT reverse transcriptase.

Quantitative PCR analysis was performed in 96-well plates on a CFX 96™ Real-Time System C1000™ Thermal Cycler (BioRad^®^) using the SYBR Green 2X (Promega^®^). Five nanograms of cDNA of each sample were processed under the following PCR conditions: one cycle of 5 min at 95°C followed by 45 cycles consisting of 10 sec at 95°C and 30 sec at 60°C. Primers used are listed in Table 1.

**Table 1:** Primers used for qPCR validation showing the accession number for each EST and the forward and reverse primers

**Gene name**	**Accession number**	**Forward primers**	**Reverse primers**
**DRAP1**	AM857148	ACTCTGAGGAGGAGGCCAAT	CCTGGTGTTGTTCATTGCTG
**RABAC**	AM867263	TTGCCAATGTAAATGCCAGA	GGAACTGGTGCCACTGACTT
**APBA2**	AM858827	ACCCGACGTCAAGTATCAGC	ATCTCGATGATTCGGTGTCC
**Activin Receptor**	AM855334	CTGGATCACACCCAACACTG	GGCTGCAACCTGCTCTAAAC
**BAR1/Bat1**	AF075691	GCCAAGTCTGGTATGGGAAA	GCCGAGGTACCAACACTGAT
**DRG2**	AM859481	TTCCCGTCTGTTGGAAAGTC	GGAATGCAGGTCAAGGTTGT
**Nup54**	AM854457	ACCCAAGCAAAAACAACAGC	CCACCAAAGGAAAATCCTGA
**PRDM5**	CU989501	TGATTCATTCAGGGATGCTG	ACATGTGGCGTTTCATGTTG
**Rac1**	CU983949	AAGCCGACAAAGAAAACGAA	TCAATGCATCATGGGAATGT
**Vsp53/Vps53**	AM866620	ATCAGCTCAGCGTCTGGTTT	TCCGACACTGTTCCTCCTCT
**GroupL1**	CU990573	CGGGATCCAAGGTTTCTGTA	ATAGGTCCCGCAAGTGTTTG
**Cbx1**	AM863306	AAGTGGTGGACTCCAGGATG	TTTGCTTCTGGTTCCCAAGT

The reference gene used in this study was EF1α (*Crassostrea gigas* EFα1 AB122066). The choice of the reference gene was determined by an expression analysis of the more stable and frequency used housekeeping genes identified by Dheilly et al. [28]. From normalized data of the microarray and also in quantitative PCR data, the coefficient of variation of GAPDH, EFα1, Actin, HKG1, Sec61, HKG3 and ARF1 was measured and was lowest for EFα1. Comparison among the four conditions of infectious level used in this study also showed that EFα1 was most stable. The validation of the selected genes by qPCR was also tested by the calculation of the coefficient of correlation between the data of microarray and qPCR.

According to their annotation and functions in each biological categories, twelve genes were chosen to assess the results from the microarray, and genes were listed in Table 1 with annotations and primers sequences.

## Results

### Percentage of virus detection associated with spat mortalities

When the spat used for this study were deployed in the field in April, 97.5% of the 120 individuals assayed for OsHV-1 were considered to be free of the virus because viral DNA could not be detected by the qPCR assay. The other 2.5% of spat contained less than 10^3^ virus genomic units (GU) ng^-1^ DNA. Moreover, mortality did not occur in these spat when held in controlled condition for one month under environmental conditions favourable for disease: high temperature (21°C) and high food levels (oysters were fed *ad libitum*) as described in Petton et al. (personal communication).

**Figure 1:**
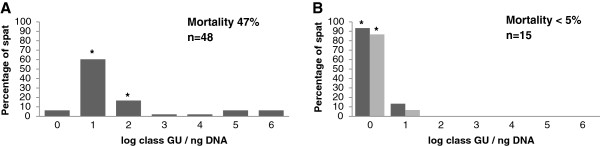
**Percentage of infected spat according to the viral load in log class.** The level of virus is expressed in genomic units (GU) per nanogram of DNA and illustrate viral load at **(A)** the field oyster growout site and in the **(B)** sanctuary site (dark grey - CRIC) and in the tank (grey – CAB)) during mortality events observed in the field. The asterisks indicate the sample taken for microarray analysis. For graphic A, the first asterik corresponds to sample infected at 4 viral GU ng^-1^ of DNA (BL), and the second refers to samples infected at 14 viralGU ng^-1^ of f DNA ( i). Data in B are from spat considered to be uninfected.

Mortality of spat located in oyster production site was observed beginning in May 2011, but only at the oyster grow out site. At the end of May spat experiencing heavy mortality were sampled and analysed individually. At this date, viral quantification showed them to be highly contaminated, with 61% and 17% in class 1 and class 2, respectively (Figure 1A). In comparison, low number of OsHV-1 DNA positive samples was observed in the sanctuary area and the containment structure, with 93 and 87%, respectively, in Class 0 (Figure 1B).

At the end of the experiment in Fall 2011, mortalities were measured and were 4.5% for tank and the sanctuary area compared to 47% for the animals sharing the same origin reared in the grow out site.

**Figure 2:**
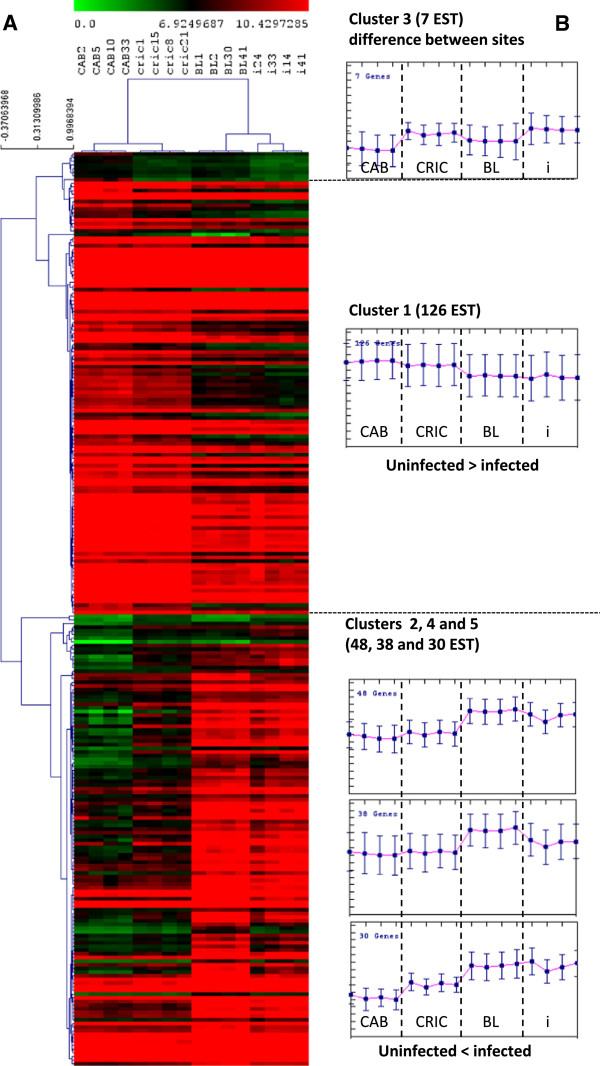
**Clustering of 249 ESTs obtained after ANOVA analysis with four groups (CAB, CRIC, BL and i – see legend to Figure**1**) with adjusted Bonferroni and p values< 0,01 made on TMeV 4.6.0 software (Saeed et al., 2003 and 2006).** CRIC and i correspond, respectively, to uninfected spat from sanctuary area or from tank. **A**: Cluster 1 is a major cluster with 126 ESTs and cluster 3 is the smaller cluster with 7 ESTs. BL and CAB correspond to infected spat from oyster field (two levels 1 and 2 log class of viral GU ng^-1^ DNA). **B**: Graphs represent values transcriptional variation of genes in each category.

From infected or non-infected animals in this experiment, a microarray analysis has been performed from 4 oysters per condition presenting a similar viral load. A test by Principal Component Analysis defined two groups with respectively together the infected animals (at two different viral loads) and the uninfected animals. A very low effect due to the different rearing sites was observed on the PCA performed on all genes (data not shown). After analysis of the microarray findings on TMeV 4.6.0 software [39,40], one-way ANOVA results of the 4 groups uninfected wild, uninfected offshore, low and high infected (respectively CRIC, CAB, BL and i) having a p value < 0.01 with Bonferroni’s correction found that 249 of 31,918 analyzed ESTs seemed to be differentially expressed (Figure 2A). A t-test (p<0.01 and Bonferroni’s correction) performed between the sanctuary and offshore sites do not show any significant expressed genes. A hierarchical clustering performed using a Pearson’s correlation defined two groups: uninfected and infected animals. A subsequent K-means clustering of genes allowed definition of 5 distinct clusters (Figure 2B). Cluster 1 is composed of 126 ESTs under expressed in infected spat. Three clusters (2, 4 and 5) grouped 48, 38 and 38 up regulated genes, respectively, in infected spat. Only 7 ESTs in Cluster 3 might be associated to site effects.

### Gene function analysis

**Figure 3:**
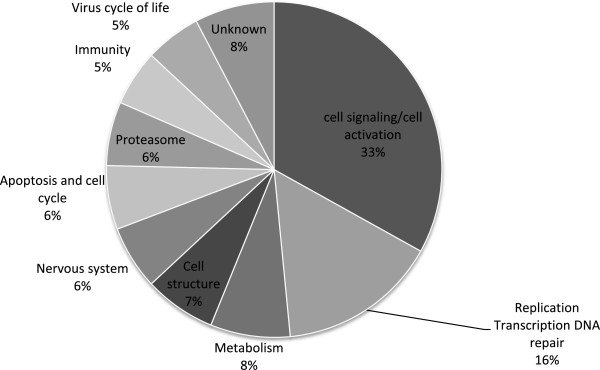
**Sector diagram representing the percentage of the 128 genes by gene function.** The list of 128 genes was obtained by comparison between infected or uninfected spat.

**Figure 4:**
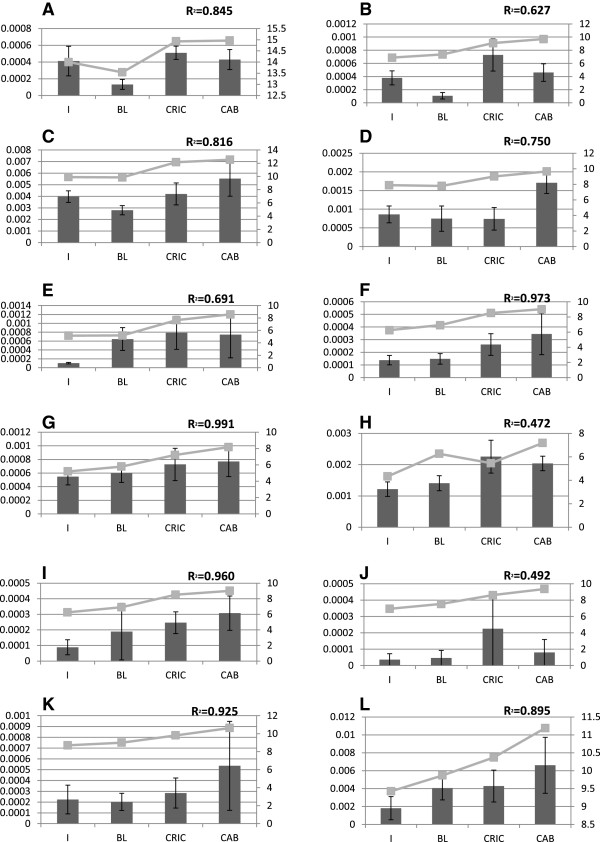
**Graph representing the profiles obtained from microarray (light grey) and qPCR (dark grey) analyses for twelve genes.** Values of qPCR are expressed in reference to EF1alpha. The same spat samples were used for both microrarry and qPCR assays. Conditions are precised in abciss and left axis represent qPCR data in DCt for100 copies of EF1alpha and right axis represent microarray data in fluorescence. R^2^ represent the correlation between microarray and qPCR data. Accession number of the genes used are BAR1: AF075691 **(A)**; Vps53: AM866620 **(B)**; RAC 1: CU983949 **(C)**; PRDM5: CU989501**(D)**; Activin receptor type-2A : AM855334 **(E)**; RABAC: AM867263 **(F)**; DRAP 1 : AM857148 **(G)**; GroupeL1: CU990573 **(H)**; ABPA2 : AM858827 **(I)**; DRG2 : AM859481 **(J)**; Nup54 : AM854457 **(K)**; Cbx1: AM863306 **(L)**.

The custom microarray design and the raw and normalized data are available from Gene Expression Omnibus (http://www.ncbi.nlm.nih.gov/geo/query/acc.cgi?acc=GSE46249; GSE46249). Only 51% of the 249 differentially expressed genes on the microarray were annotated. From the recent genome sequencing of the Pacific oyster [41], the non-annotated transcripts were blasted in genome data http://oysterdb.cn/blast.html, resulting in an additional 128 genes: 62 and 66 of genes up regulated in infected and uninfected animals, respectively (see Additional files 1 and 2). A classification of the 128 genes has been made to group different genes into their major function (Figure 3). Only 13 of the 128 genes were listed as not annotated or having an unknown function. They correspond to hypothetical proteins or non-coding region of the genome. The results from the microarray analysis were assessed by the quantification of twelve genes selected by their biological functional classification and their relative range of intensity by real time quantitative PCR (Figure 4) The coefficient of correlation between qPCR and microarray data is from 0.627 to 0.991 for 10 genes, lower for for 2 genes *groupl1* and *drg2* (respectively 0.472 and 0.492).

The major group is composed of 43 genes having various functions linked to cell signaling like *apba2*, *cib1*, *dusp7* and *adam*. The second most important group is composed of 20 genes involved in replicative and transcriptional mechanisms and regulation, or in DNA repair, including *drap1*, *bar1*/*bat1* and *nup54*. Six smaller groups of genes (with fewer than ten genes per group) are defined as genes involved in apoptosis or cell proliferation (*cbx1*, *birc7*, *drg2*, g*roupl1*) and proteasomic activities (*trim2*, *rfwd2*, *cnot4*); in immunity and inflammatory responses, including *cd109*, *activin receptor* and *leng8*; in cellular structural and architectural activities (*talin*-*2*, *actin* and different *tubulins*); in general metabolism (*fasn*, *ceruloplasmin* and *oat*); and in nervous system functions (*reticulon 4 isoform b2*, *kctd7* and *bri3*). Finally, a small group is composed of 7 host genes that could be involved directly in virus entry or in its life cycle (*prdm5*, *rabac*, *rna1 polyprotein*, *rac1*, *vps53*, *sfrS8*, *csnk2b*).

## Discussion

In order to understand the molecular events that occurred during the massive oyster mortalities, and the role played by OsHV-1 in them, animals from a same origin were reared in different areas: an oyster grow out site; a sanctuary area far from oyster growing sites and in tank isolated from natural areas. At the end of this experiment, high mortalities were observed only in the oyster grow out site and coincided with the period of repeated massive mortality events observed in France [11,14]. Due to absence of mortalities and virus in animals in the sanctuary and the isolated tank, the field-observed mortalities could be due to contamination by the virus (pathogen only measured during the experiment), which was found at high levels in oyster spat at in the grow out site. But, we cannot exclude the influence and a synergic effect of other pathogens as bacteria. In comparison to the 80-90% mortality often reported for spat since 2008, the 47% experienced by spat in our study was relatively modest, perhaps because they had been deployed only since April rather than the previous fall.

Our microarray analysis found 249 differentially expressed genes in oysters infected by OsHV-1 and in uninfected controls. Of these, approximately half (126) were underexpressed and half (116) were over-expressed in the infected spat. Only 7 were site-associated and showed differences between sites, maybe correspond to the environmental conditions. In other similar studies performed on domestic species, the number of genes differentially expressed varied from 200 to 250 [41,42]. Among the differentially expressed genes, some may or could be involved in the viral infection pathways. Others genes demonstrate the metabolic changes that occurred during the infection without exclude environmental factors between sites.

**Figure 5:**
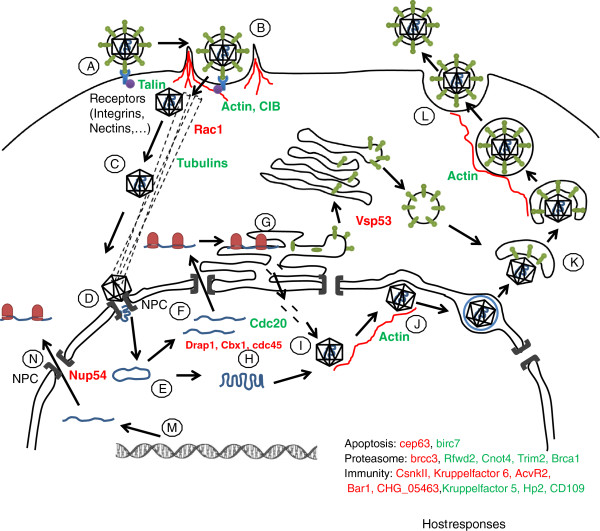
**Hypothetical scheme representing the different steps occuring during viral infection. A**: Attachment. **B**: Membrane fusion with intervention of the actin cytoskeleton and cell adhesion proteins. **C**: Translocation of capside to the nucleus with Microtubules MT. **D**: Docking at the nuclear pore: release of viral genome into the nucleus. **E**: Concatemer. **F**: Transcription of the viral mRNA. **G**: Traduction of the viral mRNA in the RER for the capsid proteins and maturation in the Golgi for the envelope proteins. **H**: Replication. **I**: Capsid formation. **J**: Budding to the inner nuclear membrane followed by release of the capsid into the cytoplasm. **K**: Envelopment of Trans Golgi network vesicule with proteins to form the envelope. **L**: Fusion of the vesicule with the virion to the plasma membrane to ensure releasing of the mature virion. **M** and **N**: hijacking cellular machinery.

From these results, we attempt to define the viral genes involved in the penetration, expression and egress processes, and also in the resulting metabolic perturbation to the host, and in the host response to the infection. Finally, we propose a hypothetical scheme of the genes expressed during the successive steps of the viral infection process and suggested by the microarray analysis (Figure 5).

### Entry of virus

First, the virus needs to bind to the cell membrane by a specific recognition of viral protein by host membrane receptors. This initial step could not be demonstrated in our analysis, involving only membrane components. Receptor type depends on the viral subfamily: the alpha herpesviridae bind to the Nectin receptor, whereas the gamma herpesviridae interact with α3β1 integrins (Figure 5A). For the latter, the linkage involves the cytoplasmic F-Actin with different proteins including Talin or Vinculin and the *talin*-*2* gene shows a higher expression in the infected animals (Figure 5B). Calcium and Integrin Binding Proteins such as CIB1 (also named Sip2-28) are also involved in the interaction with the cytoplasmic tail of the α integrin [43,44]. Tsuboi [44] suggests that it permits the activation of the integrin during the platelet aggregation. Our results suggest that the integrin pathway is activated during OsHV-1 infection in oysters, as a recognition mechanism or as a reaction of the host (Figures 5A and B).

After the virus binding to the membrane, capsid entry into the cell can follow two distinct ways. Depending on the host cell type, Herpes Simplex Virus-1 (HSV-1) can penetrate by pH-dependent endocytosis or a cortical actin-dependent, but pH independent pathway [45]. The overexpression of oyster cytoplasmic actin beta 1 and the non muscular myosin genes in infected animals could suggest the involvement of this actin pathway during the OsHV-1 infection, particularly as overexpression of these two genes was observed by Renault et al. [19] in oyster haemocytes infected by OsHV-1. In case of the actin dependent entry, the role of actin is also associated to the Rho GTPase signaling pathway including cdc42, RhoA and Rac1. Activation these three genes is host cell-type dependent and also dependent on the type of herpesvirus. Indeed, HSV-1 entry involves Rac1 and cdc42 activation in canine kidney cells (MDCKII) without RhoA, but have needs to RhoA activation in primary corneal fibroblasts [46]. Our analysis found that the *rac1* gene is under expressed in infected animals. In mouse cells, its overexpression leads to diminished capacity of the virus to enter in cells [47]. Overexpression of the Rac1 activator, as found in our study, suggests that facilitate an effective virus entry into spat cells. In their study, Renault et al. [19] demonstrated an up regulation of an EST encoding a Ras-like GTP-binding protein, Rho1, belonging to the RhoA.

### Nuclear translocation

To ensure their replication and expression, herpesvirus particles need to penetrate the nucleus after their attachment, then translocation through the cytoplasm by the cytoskeleton to the nucleus (Figure 5C). Depending on herpesvirus family and host-cell type, capsids can translocate to the nucleus through the microtubular network, but also can interact with the filamentous actin fibers, (for review Favoreel et al., [46] and [48]. In our study, α and β tubulins were overexpressed in infected animals, suggesting an involvement of these pathways capsids entry into the nucleus. Once adjacent to the nucleus, capsids dock to the nuclear envelope by the Nuclear Pore Complex (NPC), which facilitates delivery of the viral genome into the nucleoplasm. At 12h post-OsHV-1 infection, Renault et al. [19] observed an up regulation of a component of the NPC, *nup98*, in oyster haemocytes. In our study, the expression of the NPC was not up-regulated, but we showed a down-regulation of the transcript *nup54*, a nucleoporin involved with Nup62 and Nup58 to form the core nucleoporin of the NPC [49,50] have been demonstrated that a viral protein of the HSV, ICP27, is able to bind directly to the core nucleoporin Nup62 and this binding inhibits mRNA transport in the host cell. Indeed, when the viral lytic cycle begins, the HSV inactivates the host nucleoplasmic transport through the NPC by inactivation of the nucleoporin or their associated transporters.

### Viral genetic mechanisms and particle formation

Viral genetic mechanisms as replication are mediated by the virus itself. The aim of this study is to understand mechanism induced in host under viral infection. These data may give more precise information on viral replication or transcription and viral capside formation, budding and release (Figures 5E to J). Some genes like *bar1* (*bat1*) are particularly interesting in this study because of its involvement in relationship between host and pathogen [51] and this gene is already important to induced response to oxidative stress [52]. RNA-1 encoded polyprotein in viruses in plant [53] and is high expressed across infection.

### Envelope formation in the trans-golgi

Vsp53 belong to GARP complex (Golgi retrograde transport) and is involved in HIV fusion with membrane but also in virus transport in trans Golgi or lysosomes [54]. Low expression of *vsp53* in our study may avoid retrograde transport and elimination of viral particle (Figure 5K).

### Viral particle releasing or latency

Herpes viruses are able to remain latent in hosts without inducing the lytic infectious cycle [55]. Six oyster genes (*reticulon 4*, *kctd7*, *bri3*, *Smndc31*, *I*(*2*)*tid*, *cerebellin1*) are involved in nervous system. Reticulon 4 is associated with endoplasmic reticulum and inhibits axon regeneration [56]. Reticulon proteins are known to interact with human Bcl-Xl and Bcl-2 genes to reduce anti-apoptotic activity [57]. These genes may be involved in response against virus infection. KCTD7 is involved in signal conductance in the neuron [58] and is link to ubiquitin-proteasome system [59]. *Bri3* is up regulated by TNF and is link to cell death induction by TNF [60]. These six genes are down regulated in high infected spat presenting high virus DNA amount and high mortality rates, suggesting the potential for OsHV-1 to remain latent in infected oysters like some other herpes virus [61].

### Virus life cycle

Some genes are directly implicated in host response to viral infections, including *rabac*, *prdm5*, *rac1* and *vsp53*. RABAC is involved in the assembling of retro and rotavirus and in the inhibition of the herpes life cycle [62]. The under expression of this gene in infected compared to control spat suggests a failure to slow the progression of the lytic phases of the herpes virus cycle. In addition to *rabac*, the *prdm5* gene and miRNA are involved in the latency of HSV virus [63] and constituted a checkpoint for virus replication and entry into host cells in lytic phases [64]. Under expression of these two genes in infected spat strongly suggests a situation that would favour lytic phases in the virus life cycle.

### Perturbation of the host functions by the virus

Some genes involved in transcription are down regulated in our study. *Drap1* (NC2alpha) is involved in the transcriptional regulation of class II genes [65]. The down regulation of this gene may explain some disturbance in host transcription.

Eight host genes differentially expressed between non infected and infected spat are involved in proteasomic functions. Rfwd2 is an E3 ubiquitin ligase that is involved in interactions with herpes virus proteins MIR1 and 2, and act to down regulate immune recognition [66]. Cnot4 participates in the regulation of the JAK STAT signaling pathway [67] and is implicated in down regulation of TATA box [68]. Cnot4 interacts with ubiquitin conjugating enzyme E2s [69]. Tripartite motif proteins are implicated in innate immune response and some of them are involved in response to viral challenge [70].

*Rfwd2*, *cnot4* and *trim2* are up regulated in infected spat and may be involved in pseudorabies virus infection. *Ubiquitin protein ligase like* gene is involved in entry and endosomal transport of the Kaposi’s sarcoma-associated herpes virus KSHV [71]. The viral ubiquitin ligase proteins are also involved in lytic infection in HSV-1 [72]. BRCC3 is involved in ubiquitination of damaged DNA [73]. E3 ubiquitin ligase and BRCA1 are involved in ubiquitination of damaged chromosomes for genome maintenance and protein repair [74,75]. These genes are down regulated in infected spat and may be involved in the early response to the pathogen.

*Psmc4* is up regulated in infected spat and numerous PCM genes are involved in antigen processing or presentation in vertebrates (see Jenner and Young, for review [76]). Moreover, overexpression of a gene presenting homologies with IK cytokine genes has been reported in *C*. *gigas* haemocytes after OsHV-1 challenge [19].

In our study, some differentially genes expressed were involved in the global metabolism of host cells. Similarly, in salmon infected by Orthomyxovirus, differentially expressed genes were mainly down regulated and are those involved in biosynthesis and metabolism [42]. The ceruloplasmin level increased during herpes infection and decreased during remission in human [77]. During herpes infections, many genes involved in metabolism like *oat* or *fasn* are under expressed, whereas others, like *dbI* and *wnk1*, are overexpressed, suggesting a disruption in host metabolic activity.

### Host responses to the virus

#### Apoptosis and Cell cycle

*Cbx1* is involved in mitosis but also in epigenetic repression and control of mitotis [78,79]. Cdc20 and cdc45 are involved in mitosis as well [80,81]. Cbx1 and cdc45 were down regulated, but *cdc20* was upregulated, in infected spat. DRG2, also under-expressed, is involved in cellular proliferation [82] and is induced by rhabdovirus in fish [83]. This gene is known to play a key role in cell growth [84], and the downregulation of this gene may reduce cell growth and division. The genes, *nedd1* and *cep63* are downregulated in infected spat and these genes are associated to centrosome formation [85,86]. *Birc7*, a gene involved in the inhibition of apoptosis, is upregulated in infected spat [87], as is *groupl1*, which is concerned with the regulation of genes involved in anti-apoptotic mechanisms [37]. Overexpression of genes related to apoptosis such as Bcl-2 (GenBank accession EU678310) have been reported in *C*. *gigas* haemocytes after OsHV-1 challenge [19].

Three of these genes (*cbx1*, *drg2*, *groupl1*) have been validated by qPCR (Figure 3) suggesting that cellular proliferation may be reduced in infected spat. Anti-apoptosis phenomena appear to be important in infected spat. It may exist a balance between pro apoptosis and anti-apoptosis factors suggest by Jenner and Young in 2005 (for review [76]).

#### Immune system

Seven genes are linked to immunity function. Two of them, which were overexpressed in infected spat, are associated with the response to fungal infection, such as *hlp2* and a putative fungi-static metabolite, CHFF_05463. *Leng8* is involved in regulation of the inflammation process and these receptor present homologues in herpes saimiri [88]. CD109 represents an evolutionary conserved class of α2-macroglobulin/complement gene family [38] and is involved in regulation of the TGFbeta pathway [88]. The α2-macroglobulin molecules are protease inhibitors [89]. In *C*. *gigas*, ESTs from hemocytes show high identity with α2-macroglobulin and its receptor [90]. These genes are overexpressed in infected spat and suggest a role in defense against the pathogen. Moreover, in SSH studies overexpression of a gene presenting homologies with alpha macroglobuline genes (GenBank accession EU678312) has been reported in *C*. *gigas* haemocytes after OsHV-1 challenge [19].

Hemicentin HMCN1 is a conserved extracellular member of the immunoglobulin superfamily [91]. The activin receptor is involved in activation of heterologous herpes simplex virus thymidine kinase [92] and plays a role in response to herpes type 8 [93]. Moreover activin A is involved in APRIL regulation and leads to stimulation of immune cells [94]. These two genes are downregulated in late infected spat and may be expressed at the beginning of the response to infection.

#### Cell signalling

Forty-three genes found to be differentially expressed in our study are link to cell signaling or cell activation. They comprised the major group of the gene identified. Some of them are associated to specific pathways like Mapk (for Dusp7) or PI3K (as well as Grb2, PKN2). *Dusp7* is downregulated in infected spat. DUSP (Dual Specificity Phosphatase) proteins are phosphatases that limit the immune response and are linked with apoptosis activation if the infection progresses into one considered to be of “high-alert” [76]. Down regulation of *dusp7* might lead to reduced apoptosis or to stimulate the immune response in infected spat.

ADAM proteins are involved in adhesion and protease activity, and some of them are membrane bound enzymes able to cleave transmembrane proteins and named sheddases (Primakoff and Myles for review [95]). The genes *adam* and adamTS16, down-regulated in infected spat, might be involved in cellular response to infection. Other studies showed decreased expression in chicken embryo lung cells infected with virus [96].

The down regulation of *apba2* suggested a decrease in the cell activity in infected spat. APBA2 is implicated in exocytosis of synaptic vesicles [97] and indicated that traffic cellular organelles decreased.

The *Armadillo* gene was up regulated in infected spat and some authors hypothesized a role of Armadillo in TLR signalling [98].

In our study, genes differentially expressed across pathogenesis are involved in many physiological process and we tried to propose hypothetic scheme as preliminary results, but infectious disease is the result of complex interactions between a host and its pathogen involving a lot of process, not only related to immunity. These results seem to confirm the involvement of the virus in mortality and allowed us to propose a hypothetical scenario to explain the different steps of the viral infectious cycle during the massive spat mortalities. Nevertheless, this pathogen is probably not the only agent involved in the mortality, as other evidence suggests a role for bacteria [99]. This leads to the possibility that a relationship between bacteria and the virus across pathogenesis.

It would be very interesting to follow the infection OsHV-1 infection process with a viral challenge made under controlled conditions in order to document the kinetics of the expression profile as the infection proceeds. The limit of this study is the use of natural sites and controlled conditions may exclude environment parameters and reduce effect of multipara metric factors. Such an experiment would lead to a better understanding of the responses by infected oysters against the virus and to be more precised on pathways involving virus and host during early infection. Moreover it may be useful to use survivors as model to understand why some oysters are resistant to the virus and why some of them die. A study of this sort might even provide a view of resistance mechanisms developed by oysters to pathogens in general.

It might be interesting too to follow the response of viral genome during infectious challenge with a mixed microarray containing viral and oyster genomes. Such a study may permit to reach viral replication and multiplication mechanisms and the control between lytic phase and latency.

To conclude, we have proposed an OsHV-1 – oyster infection scenario according to genome annotation and these results show transcriptomic regulation involved in response against virus. The ability to regulate the OsHV-1 genome activity may allow some oysters to survive infection.

## Competing interests

Non-financial competing interests.

## Authors’ contributions

AJ carried out the molecular biology aspects including microarrays and qPCR validation. ML participated to DNA quantification in virus load detection. JLB and MH participated to spat qualification. CL and AJ participated to perform the statistical analysis and the interpretation of microarray data. AJ, MM and CL conceived of the study, and participated in its design and coordination and helped to draft the manuscript. All authors read and approved the final manuscript.

## Supplementary Material

Addition file 1 **List of 66 genes upregulated in non infected spat obtained after ANOVA analysis with four groups (CAB, CRIC, BL an i) with p value< 0,01 and adjusted Bonferroni on TMeV 4.6.0 software (Saeed et al. 2003 and 2006).** The accession number, description from the *C*. *gigas* database or oyster genome, R^2^, other names used for the gene is provided for each gene. Click here for file

Addition file 2 **List of 62 genes upregulated in infected spat obtained after ANOVA analysis with four groups (CAB, CRIC, BL an i) with p value< 0,01 and adjusted Bonferroni on TMeV 4.6.0 software (Saeed et al. 2003 and 2006).** The accession number, description from the *C*. *gigas* database or oyster genome, R^2^, other names used for the gene is provided for each gene. Click here for file
