# Supplementary material for: Physiological change under OsHV-1 contamination in Pacific oyster Crassostrea gigas through massive mortality events on fields
Source: BMC Genomics. 2013 Aug 29;14:590. doi: 10.1186/1471-2164-14-590 (PMC3766697; doi:10.1186/1471-2164-14-590)
Supplement: Addition file 1 — List of 66 genes upregulated in non infected spat obtained after ANOVA analysis with four groups (CAB, CRIC, BL an i) with p value< 0,01 and adjusted Bonferroni on TMeV 4.6.0 software (Saeed et al. 2003 and 2006). The accession number, description from the C. gigas database or oyster genome, R2, other names used for the gene is provided for each gene. [file 1471-2164-14-590-S1.pptx]

## Slide 1
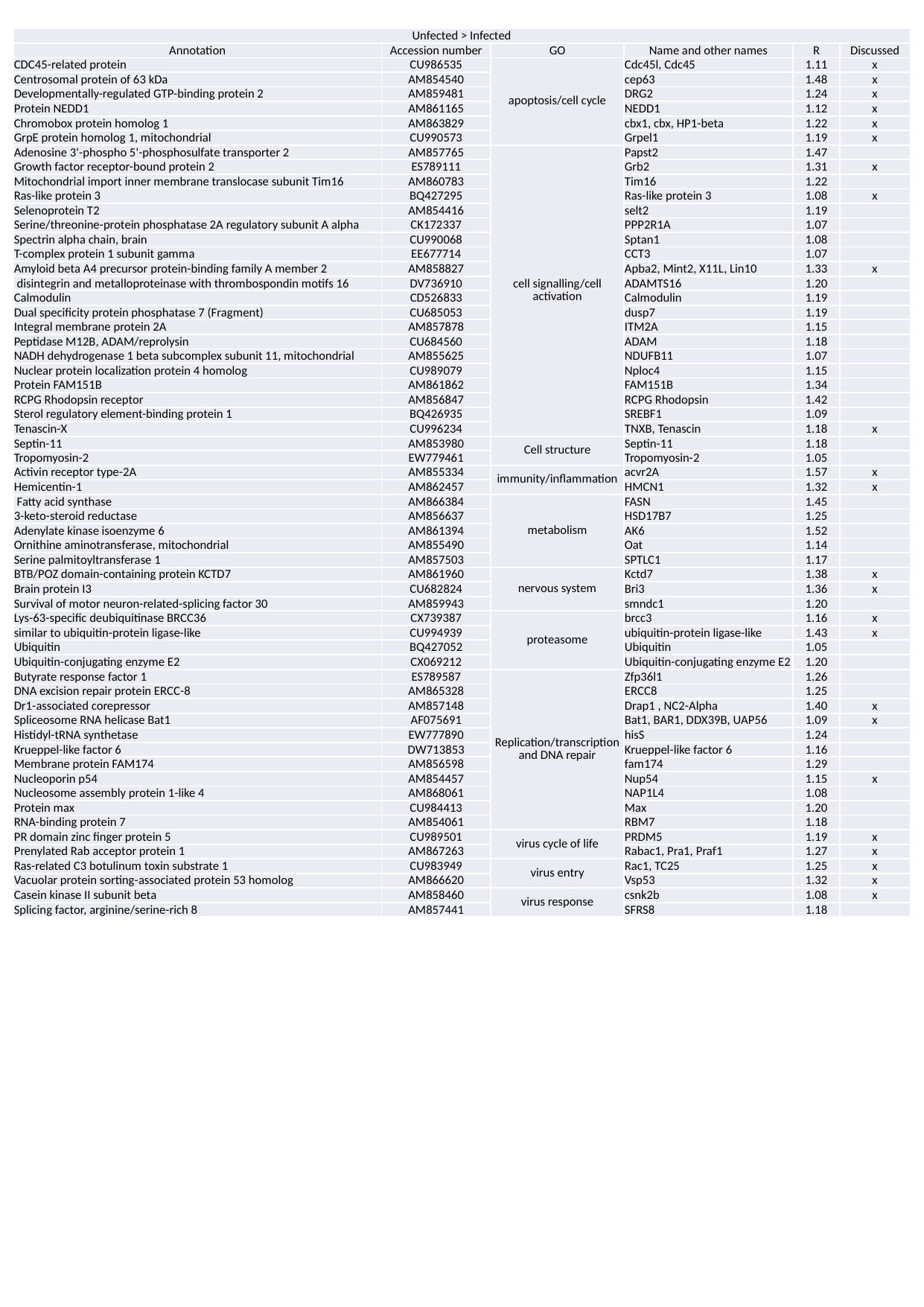

| Unfected > Infected | | | | | |
| --- | --- | --- | --- | --- | --- |
| Annotation | Accession number | GO | Name and other names | R | Discussed |
| CDC45-related protein | CU986535 | apoptosis/cell cycle | Cdc45l, Cdc45 | 1.11 | x |
| Centrosomal protein of 63 kDa | AM854540 | | cep63 | 1.48 | x |
| Developmentally-regulated GTP-binding protein 2 | AM859481 | | DRG2 | 1.24 | x |
| Protein NEDD1 | AM861165 | | NEDD1 | 1.12 | x |
| Chromobox protein homolog 1 | AM863829 | | cbx1, cbx, HP1-beta | 1.22 | x |
| GrpE protein homolog 1, mitochondrial | CU990573 | | Grpel1 | 1.19 | x |
| Adenosine 3'-phospho 5'-phosphosulfate transporter 2 | AM857765 | cell signalling/cell activation | Papst2 | 1.47 | |
| Growth factor receptor-bound protein 2 | ES789111 | | Grb2 | 1.31 | x |
| Mitochondrial import inner membrane translocase subunit Tim16 | AM860783 | | Tim16 | 1.22 | |
| Ras-like protein 3 | BQ427295 | | Ras-like protein 3 | 1.08 | x |
| Selenoprotein T2 | AM854416 | | selt2 | 1.19 | |
| Serine/threonine-protein phosphatase 2A regulatory subunit A alpha | CK172337 | | PPP2R1A | 1.07 | |
| Spectrin alpha chain, brain | CU990068 | | Sptan1 | 1.08 | |
| T-complex protein 1 subunit gamma | EE677714 | | CCT3 | 1.07 | |
| Amyloid beta A4 precursor protein-binding family A member 2 | AM858827 | | Apba2, Mint2, X11L, Lin10 | 1.33 | x |
| disintegrin and metalloproteinase with thrombospondin motifs 16 | DV736910 | | ADAMTS16 | 1.20 | |
| Calmodulin | CD526833 | | Calmodulin | 1.19 | |
| Dual specificity protein phosphatase 7 (Fragment) | CU685053 | | dusp7 | 1.19 | |
| Integral membrane protein 2A | AM857878 | | ITM2A | 1.15 | |
| Peptidase M12B, ADAM/reprolysin | CU684560 | | ADAM | 1.18 | |
| NADH dehydrogenase 1 beta subcomplex subunit 11, mitochondrial | AM855625 | | NDUFB11 | 1.07 | |
| Nuclear protein localization protein 4 homolog | CU989079 | | Nploc4 | 1.15 | |
| Protein FAM151B | AM861862 | | FAM151B | 1.34 | |
| RCPG Rhodopsin receptor | AM856847 | | RCPG Rhodopsin | 1.42 | |
| Sterol regulatory element-binding protein 1 | BQ426935 | | SREBF1 | 1.09 | |
| Tenascin-X | CU996234 | | TNXB, Tenascin | 1.18 | x |
| Septin-11 | AM853980 | Cell structure | Septin-11 | 1.18 | |
| Tropomyosin-2 | EW779461 | | Tropomyosin-2 | 1.05 | |
| Activin receptor type-2A | AM855334 | immunity/inflammation | acvr2A | 1.57 | x |
| Hemicentin-1 | AM862457 | | HMCN1 | 1.32 | x |
| Fatty acid synthase | AM866384 | metabolism | FASN | 1.45 | |
| 3-keto-steroid reductase | AM856637 | | HSD17B7 | 1.25 | |
| Adenylate kinase isoenzyme 6 | AM861394 | | AK6 | 1.52 | |
| Ornithine aminotransferase, mitochondrial | AM855490 | | Oat | 1.14 | |
| Serine palmitoyltransferase 1 | AM857503 | | SPTLC1 | 1.17 | |
| BTB/POZ domain-containing protein KCTD7 | AM861960 | nervous system | Kctd7 | 1.38 | x |
| Brain protein I3 | CU682824 | | Bri3 | 1.36 | x |
| Survival of motor neuron-related-splicing factor 30 | AM859943 | | smndc1 | 1.20 | |
| Lys-63-specific deubiquitinase BRCC36 | CX739387 | proteasome | brcc3 | 1.16 | x |
| similar to ubiquitin-protein ligase-like | CU994939 | | ubiquitin-protein ligase-like | 1.43 | x |
| Ubiquitin | BQ427052 | | Ubiquitin | 1.05 | |
| Ubiquitin-conjugating enzyme E2 | CX069212 | | Ubiquitin-conjugating enzyme E2 | 1.20 | |
| Butyrate response factor 1 | ES789587 | Replication/transcription and DNA repair | Zfp36l1 | 1.26 | |
| DNA excision repair protein ERCC-8 | AM865328 | | ERCC8 | 1.25 | |
| Dr1-associated corepressor | AM857148 | | Drap1 , NC2-Alpha | 1.40 | x |
| Spliceosome RNA helicase Bat1 | AF075691 | | Bat1, BAR1, DDX39B, UAP56 | 1.09 | x |
| Histidyl-tRNA synthetase | EW777890 | | hisS | 1.24 | |
| Krueppel-like factor 6 | DW713853 | | Krueppel-like factor 6 | 1.16 | |
| Membrane protein FAM174 | AM856598 | | fam174 | 1.29 | |
| Nucleoporin p54 | AM854457 | | Nup54 | 1.15 | x |
| Nucleosome assembly protein 1-like 4 | AM868061 | | NAP1L4 | 1.08 | |
| Protein max | CU984413 | | Max | 1.20 | |
| RNA-binding protein 7 | AM854061 | | RBM7 | 1.18 | |
| PR domain zinc finger protein 5 | CU989501 | virus cycle of life | PRDM5 | 1.19 | x |
| Prenylated Rab acceptor protein 1 | AM867263 | | Rabac1, Pra1, Praf1 | 1.27 | x |
| Ras-related C3 botulinum toxin substrate 1 | CU983949 | virus entry | Rac1, TC25 | 1.25 | x |
| Vacuolar protein sorting-associated protein 53 homolog | AM866620 | | Vsp53 | 1.32 | x |
| Casein kinase II subunit beta | AM858460 | virus response | csnk2b | 1.08 | x |
| Splicing factor, arginine/serine-rich 8 | AM857441 | | SFRS8 | 1.18 | |
