# Supplementary material for: Physiological change under OsHV-1 contamination in Pacific oyster Crassostrea gigas through massive mortality events on fields
Source: BMC Genomics. 2013 Aug 29;14:590. doi: 10.1186/1471-2164-14-590 (PMC3766697; doi:10.1186/1471-2164-14-590)
Supplement: Addition file 2 — List of 62 genes upregulated in infected spat obtained after ANOVA analysis with four groups (CAB, CRIC, BL an i) with p value< 0,01 and adjusted Bonferroni on TMeV 4.6.0 software (Saeed et al. 2003 and 2006). The accession number, description from the C. gigas database or oyster genome, R2, other names used for the gene is provided for each gene. [file 1471-2164-14-590-S2.pptx]

## Slide 1
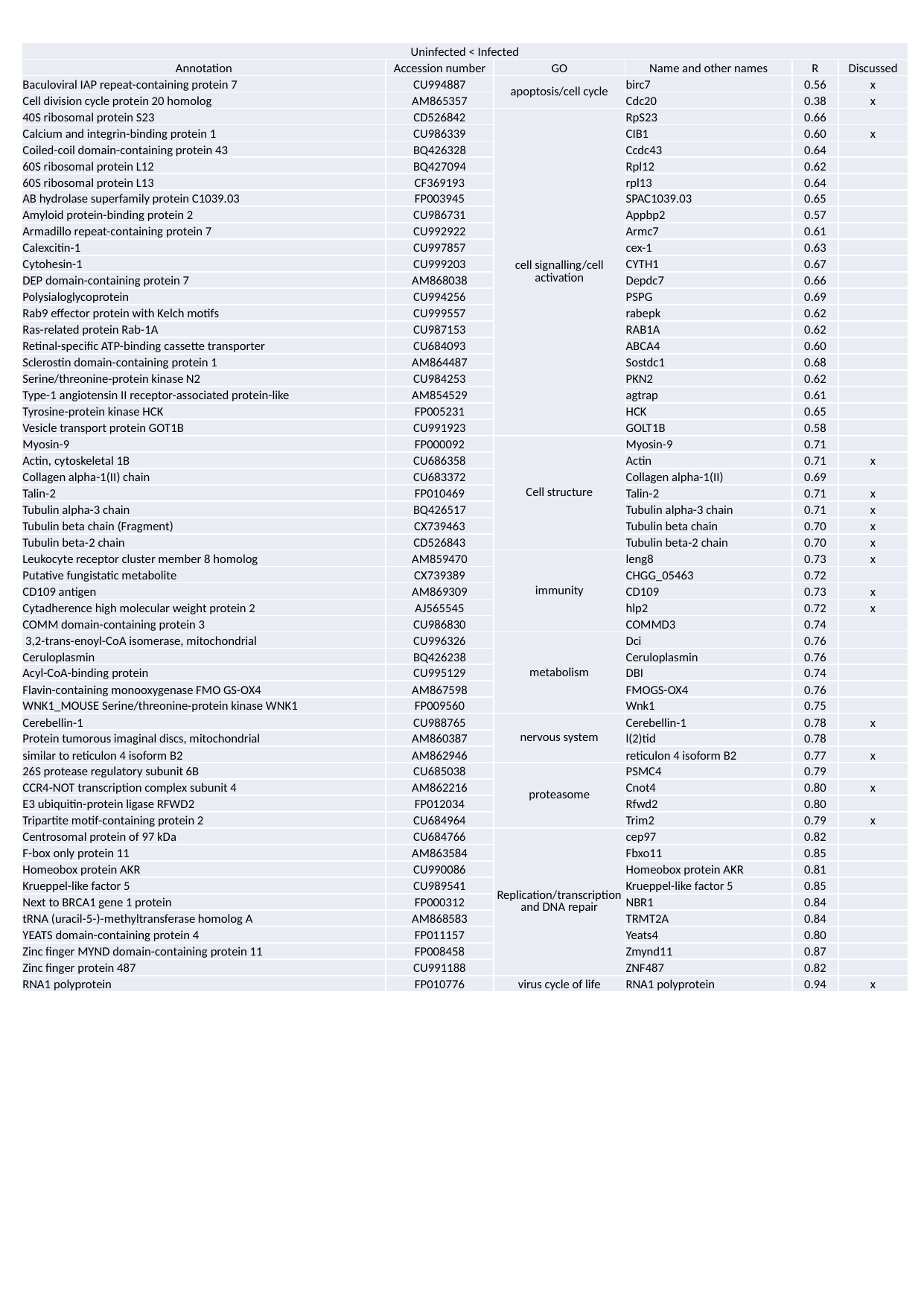

| Uninfected < Infected | | | | | |
| --- | --- | --- | --- | --- | --- |
| Annotation | Accession number | GO | Name and other names | R | Discussed |
| Baculoviral IAP repeat-containing protein 7 | CU994887 | apoptosis/cell cycle | birc7 | 0.56 | x |
| Cell division cycle protein 20 homolog | AM865357 | | Cdc20 | 0.38 | x |
| 40S ribosomal protein S23 | CD526842 | cell signalling/cell activation | RpS23 | 0.66 | |
| Calcium and integrin-binding protein 1 | CU986339 | | CIB1 | 0.60 | x |
| Coiled-coil domain-containing protein 43 | BQ426328 | | Ccdc43 | 0.64 | |
| 60S ribosomal protein L12 | BQ427094 | | Rpl12 | 0.62 | |
| 60S ribosomal protein L13 | CF369193 | | rpl13 | 0.64 | |
| AB hydrolase superfamily protein C1039.03 | FP003945 | | SPAC1039.03 | 0.65 | |
| Amyloid protein-binding protein 2 | CU986731 | | Appbp2 | 0.57 | |
| Armadillo repeat-containing protein 7 | CU992922 | | Armc7 | 0.61 | |
| Calexcitin-1 | CU997857 | | cex-1 | 0.63 | |
| Cytohesin-1 | CU999203 | | CYTH1 | 0.67 | |
| DEP domain-containing protein 7 | AM868038 | | Depdc7 | 0.66 | |
| Polysialoglycoprotein | CU994256 | | PSPG | 0.69 | |
| Rab9 effector protein with Kelch motifs | CU999557 | | rabepk | 0.62 | |
| Ras-related protein Rab-1A | CU987153 | | RAB1A | 0.62 | |
| Retinal-specific ATP-binding cassette transporter | CU684093 | | ABCA4 | 0.60 | |
| Sclerostin domain-containing protein 1 | AM864487 | | Sostdc1 | 0.68 | |
| Serine/threonine-protein kinase N2 | CU984253 | | PKN2 | 0.62 | |
| Type-1 angiotensin II receptor-associated protein-like | AM854529 | | agtrap | 0.61 | |
| Tyrosine-protein kinase HCK | FP005231 | | HCK | 0.65 | |
| Vesicle transport protein GOT1B | CU991923 | | GOLT1B | 0.58 | |
| Myosin-9 | FP000092 | Cell structure | Myosin-9 | 0.71 | |
| Actin, cytoskeletal 1B | CU686358 | | Actin | 0.71 | x |
| Collagen alpha-1(II) chain | CU683372 | | Collagen alpha-1(II) | 0.69 | |
| Talin-2 | FP010469 | | Talin-2 | 0.71 | x |
| Tubulin alpha-3 chain | BQ426517 | | Tubulin alpha-3 chain | 0.71 | x |
| Tubulin beta chain (Fragment) | CX739463 | | Tubulin beta chain | 0.70 | x |
| Tubulin beta-2 chain | CD526843 | | Tubulin beta-2 chain | 0.70 | x |
| Leukocyte receptor cluster member 8 homolog | AM859470 | immunity | leng8 | 0.73 | x |
| Putative fungistatic metabolite | CX739389 | | CHGG\_05463 | 0.72 | |
| CD109 antigen | AM869309 | | CD109 | 0.73 | x |
| Cytadherence high molecular weight protein 2 | AJ565545 | | hlp2 | 0.72 | x |
| COMM domain-containing protein 3 | CU986830 | | COMMD3 | 0.74 | |
| 3,2-trans-enoyl-CoA isomerase, mitochondrial | CU996326 | metabolism | Dci | 0.76 | |
| Ceruloplasmin | BQ426238 | | Ceruloplasmin | 0.76 | |
| Acyl-CoA-binding protein | CU995129 | | DBI | 0.74 | |
| Flavin-containing monooxygenase FMO GS-OX4 | AM867598 | | FMOGS-OX4 | 0.76 | |
| WNK1\_MOUSE Serine/threonine-protein kinase WNK1 | FP009560 | | Wnk1 | 0.75 | |
| Cerebellin-1 | CU988765 | nervous system | Cerebellin-1 | 0.78 | x |
| Protein tumorous imaginal discs, mitochondrial | AM860387 | | l(2)tid | 0.78 | |
| similar to reticulon 4 isoform B2 | AM862946 | | reticulon 4 isoform B2 | 0.77 | x |
| 26S protease regulatory subunit 6B | CU685038 | proteasome | PSMC4 | 0.79 | |
| CCR4-NOT transcription complex subunit 4 | AM862216 | | Cnot4 | 0.80 | x |
| E3 ubiquitin-protein ligase RFWD2 | FP012034 | | Rfwd2 | 0.80 | |
| Tripartite motif-containing protein 2 | CU684964 | | Trim2 | 0.79 | x |
| Centrosomal protein of 97 kDa | CU684766 | Replication/transcription and DNA repair | cep97 | 0.82 | |
| F-box only protein 11 | AM863584 | | Fbxo11 | 0.85 | |
| Homeobox protein AKR | CU990086 | | Homeobox protein AKR | 0.81 | |
| Krueppel-like factor 5 | CU989541 | | Krueppel-like factor 5 | 0.85 | |
| Next to BRCA1 gene 1 protein | FP000312 | | NBR1 | 0.84 | |
| tRNA (uracil-5-)-methyltransferase homolog A | AM868583 | | TRMT2A | 0.84 | |
| YEATS domain-containing protein 4 | FP011157 | | Yeats4 | 0.80 | |
| Zinc finger MYND domain-containing protein 11 | FP008458 | | Zmynd11 | 0.87 | |
| Zinc finger protein 487 | CU991188 | | ZNF487 | 0.82 | |
| RNA1 polyprotein | FP010776 | virus cycle of life | RNA1 polyprotein | 0.94 | x |
